# Preoperative assessment for minimally invasive lung surgery: Need an update?

**DOI:** 10.1111/1759-7714.13753

**Published:** 2020-11-19

**Authors:** Fairuz Boujibar, Francis‐Edouard Gravier, Jean Selim, Jean‐Marc Baste

**Affiliations:** ^1^ Department of General and Thoracic Surgery CHU Rouen Rouen France; ^2^ INSERM U1096, CHU Rouen Rouen France; ^3^ ADIR Association Rouen France; ^4^ UPRES EA380 CHU Rouen Rouen France; ^5^ Department of Anesthesiology and Critical Care Rouen University Hospital Rouen France

Preoperative assessment before lung resection could confirm patients' operability and reduce mortality and morbidity.

There has been a continuous improvement in surgical technology over the past twenty years. Surgical approaches are less invasive and patients undergo videoscopic or robotic assisted thoracic surgery (VATS or RATS) which is associated with the latest advances in terms of anesthesia and pain management. Current evidence shows fewer complications, shorter length of hospital stay and less postoperative pain^1^ with these minimally invasive techniques. Preoperative time is now associated with patient optimization and prehabilitation programs, which have clearly improved postoperative outcomes. Patient outcome after thoracic surgery have changed, but not the way in which these patients are assessed preoperatively.

Over the last few years there has been a focus on preoperative spirometric evaluation. A cardiopulmonary exercise test (CPET) is recommended when the postoperative forced expiratory volume in one second (FEV1_PPO_) and/or diffusing capacity for carbon monoxide (DLCO_PPO_) are less than 30% of the predicted value, or when the performance of the stair‐climbing test or the incremental shuttle walk test is insufficient. Patients under the peak oxygen consumption (V˙O_2peak_) threshold value of 10 mL/kg/minute are associated with a high risk of perioperative morbi‐mortality, whereas those with a V˙O_2peak_ over 20 mL/kg/minute are associated with a low risk of postoperative complications.^2^

The meta‐analysis by Benzo *et al*. published in 2008 established that the perioperative predictive nature of the V˙O_2peak_ was central to the identification of these threshold values.^3^ However, all the patients included in this meta‐analysis had a thoracotomy between 1985 and 2004. In consequence, current recommendations are based on patients who did not benefit from the more recent and less invasive surgical techniques detailed above.

In reality, these recommendations are poorly followed, with only 0.5% of surgeons complying with ACCP guidelines; 4.4% were 75% adherent to ACCP guidelines and 45.8% were 50% adherent.^4^

An update on preoperative assessment is long awaited. Currently, very few studies are available on the preoperative assessment of patients who receive only minimally invasive surgery. First, we highlight a study published in 2017^5^ which evaluated the predictive performances of the incremental shuttle walking test. The authors concluded that patients walking more than 400 meters had a very low incidence of complications and would not require CPET prior to lung resection. Second, Benattia *et al*.^6^ concluded that FEV1, but not DLCO, was a significant predictor of pulmonary complications after VATS. Conversely, in the study by Berry *et al*.^7^ preoperative pulmonary function tests (DLCO and FEV1) were found to be predictors of pulmonary complications when lobectomy for lung cancer was performed through thoracotomy, but not through thoracoscopy.

In a consensus statement, a panel of 50 experts representing institutions with considerable experience in performing VATS lobectomy procedures contraindicated VATS if FEV1 and/or DLCO values were less than 30% predicted.^8^ A recent study determined this threshold at 50% for RATS resection,^9^ Whilst our report is retrospective, it does represent the largest study to date to examine the predictive value of FEV1 and DLCO for patients undergoing robotic lobectomy.

A study published by Kouritas *et al*.^10^ shows that a wider lung parenchymal resection than preoperatively planned may be performed by VATS, even in patients with low lung function (ppoFEV1 and ppoDLCO under 40%) and functional status. The authors do not report any adverse outcomes in these patients when compared to patients with good lung function, and 23/73 (31.5%) patients had a higher resection than planned (bilobectomy and pneumonectomy instead of lobectomy) and pulmonary morbidity between groups (planned vs. nonplanned resection) were similar (*P* = 0.88). They question the stratification of preoperative risk based on lung function tests in the particularly on minimally invasive approach.

We are currently unable to find any studies which evaluate the predictive nature of VO_2peak_ in patients where surgery has been performed only with the surgical assistance of VATS or RATS.

To answer our initial question regarding the need for an update, we believe that more research on preoperative assessment methods in patients who have undergone minimally invasive surgery is necessary. Indeed, the current preoperative decision algorithm may benefit from an update. More tools should be developed to screen high‐risk patients, even with minimally invasive techniques, in order to refer them to specific prehabilitation programs or nonsurgical treatments.

## Disclosure

No authors report any conflict of interest.
